# Editorial

**Published:** 2016-08-05

**Authors:** Tarek Shaarawy

**Affiliations:** Editor-in-Chief, University of Geneva, Switzerland

**The Business of Glaucoma Surgery and the Era of Mega-interest**

For decades, glaucoma therapy innovations have been a strategic goal for many pharmaceutical industry giants. A chronic disease that spans a lifetime, with most patients destined to eye drops applied on a daily basis, glaucoma was and still is a disease from which profits can be made. Glaucoma surgery, on the contrary, was but a small part of this immense business, estimated to be in the range of 5% of the whole glaucoma diagnosis and treatment market. To this day, trabeculectomy remains the gold standard procedure for surgical management of glaucoma. A procedure that requires only a few instruments, wherein one corner of a shaving blade mounted on a razor fragment holder can still be the only knife needed.

Even with the dawn of the era of wound healing manipulation, molecules used were not patented specifically for ophthalmic care, and a fraction of a volume of a vial of such drugs was sufficient to treat many patients. Glaucoma drainage devices, on the contrary, never dethroned trabeculectomy and remain a surgery reserved for refractory cases, rarely attempted as a primary procedure. A quarter of a century ago, nonpenetrating surgery was introduced, and even that, a totally new genre of procedures, did not quite change the business of glaucoma surgery. It remained a niche surgery, practiced by a minority group of glaucoma specialists, and even those did not agree among themselves on the necessity of using the handful of implants that were FDA approved or CE marked.

While glaucoma surgeons watched by as cataract, refractive, and retina surgeries evolved into something significantly more sophisticated, it became clear that there was a real need to look beyond what was available and make use of medical advancements achieved in other fields so as to offer better surgical solutions to the glaucoma patient. Trabeculectomy efficacy was not disputed, but its complication rates were equally traumatizing to patient and surgeon alike. Such complications put surgery as a last resort in the hands of most specialists, coming a distant third after medical and laser therapy. This was certainly the stimulus that prompted researchers to look for safer procedures and was first attempted by avoiding penetration and later by minimally penetrating.

New startups started to spring up from multiple incubators, many benefiting from technologies that have been tried and tested in coronary vascular procedures and other bypass surgeries. This ushered in a new era in the business of glaucoma surgery. The new devices were not widely commercialized by their producing companies, preferring to build a steady scientific base of well-designed published trials before doing so. It is fair to assume that most of these companies had their eyes set from the very start on a bigger prize, namely, to be purchased by a mega company. Thus, a new era was on the horizon.

The opening move was made by Alcon (Fort Worth, Texas), purchasing Optonol Ltd. (Neve Ilan, Israel) in 2009. With its significant marketing muscle, it was able to exponentially increase its sales worldwide, and for the first time, make a sizable dent in trabeculectomy numbers, achieving 22% of the US trabeculectomy figures, according to published company sources.

Recently, Allergan plc. (Dublin, Ireland) acquired AqueSys, Inc. (California, USA) for more than 300 million US$. Alcon shortly followed by acquiring Transcend Medical, Inc. (California, USA) and strengthening its portfolio with a suprachoroidal shunt. Other major players from the pharmaceutical industry are probably weighing their options as these words are being typed.

For the ophthalmic surgeon, this newfound interest in glaucoma surgery by the mega pharmaceutical industry has to be managed prudently. It most certainly provides the opportunity, as well as merits caution.

What the mega companies will bring to glaucoma surgery is obvious. With their vast resources, the industry would support better research and development, ensure a wider supply chain, and in due time, introduce improvements. This will also increase their appetite for more investment in glaucoma surgery as they would, understandably, aim for market size and market share growth. On the contrary, ophthalmic surgeons will have many hands knocking on their doors, with more options to contend with, and with that, a responsibility to deliberate on the tough choices that would have significant impact on the quality of life and well-being of the patient. To this end, the Journal of Current Glaucoma Practice will take every opportunity to present itself to the ophthalmologist and glaucoma specialist as a trusted partner providing unbiased high-quality information, thus fulfilling its goals and aspirations.

Tarek Shaarawy

**Editor-in-Chief**

University of Geneva, Switzerland

